# The Poison of Typhoid Fever Conveyed by Water

**Published:** 1878-02

**Authors:** 


### The Poison of Typhoid Fever Conveyed by Water.

Local epidemics of typhoid caused by the evacuation of a patient gaining access to drinking water are well worthy of being placed on record. A recent number of the *Berliner Klinsche Wochenschrift* contains an account of five cases in which infected water was the undoubted medium of communication. A miller, living on the bank of a small stream, was attacked by typhoid. His excreta were thrown daily into a small pond, which was connected with the stream by a ditch. The man recovered, but, seven weeks after his attack, his brother, who alone had nursed him, fell ill with serious symptoms of typhoid. The discharges from the bowels were very profuse, and were, as before, thrown into the pond. A few days afterwards four workmen, who were employed at a forge about a mile distant, were also attacked by typhoid, and the medical attendant, anxious to discover the source of the mischief, found that all these men obtained their drinking water from the stream in question, there being no well available. Another case also occurred, the patient being a farm labourer who worked in a meadow near the forge, and who also drank the water from the stream. No case of typhoid had been known to occur in the neighborhood for a very long time previously, and all circumstances were in favour of the supposition that the disease had been transmitted through the medium of the water. There was no evidence as to the source of infection in the case of the first patient. The period of incubation was extraordinarily long, particularly in the case of the brother. The history of this small epidemic tends to support Dr. W. Budd's view, that a specific agent must be present for the production of typhoid fever. Probably the water of the brook in question often contained more or less ordinary fecal matter. The lesson to be drawn is that the excreta of typhoid patients should be completely disinfected, and, where possible, as in country places, should be deeply buried in spots far removed from any source of water supply.*—*Med. Examiner*, Nov. 29, 1877.

*Prof. Austin Flint first demonstrated that Typhoid Fever was transmitted through the medium of water and published the observation in Buffalo Medical Journal.

